# Whole-cortical graphical networks at wakeful rest in young and older adults revealed by functional near-infrared spectroscopy

**DOI:** 10.1117/1.NPh.5.3.035004

**Published:** 2018-07-27

**Authors:** Lin Li, Olajide Babawale, Amarnath Yennu, Cynthia Trowbridge, Ryan Hulla, Robert J. Gatchel, Hanli Liu

**Affiliations:** a University of Texas at Arlington, Department of Bioengineering and Joint Graduate Program Between University of Texas at Arlington and University of Texas Southwestern Medical Center, Arlington, Texas, United States; b University of California at Los Angeles, David Geffen School of Medicine, Department of Neurology, Los Angeles, California, United States; c Stanford University School of Medicine, Department of Neurology, Stanford, California, United States; d University of Texas at Arlington, Department of Kinesiology, Arlington, Texas, United States; e University of Texas at Arlington, College of Science, Department of Psychology, Arlington, Texas, United States

**Keywords:** functional brain networks, graph theory, aging, young and older adults

## Abstract

A good understanding of age-dependent changes and modifications in brain networks is crucial for fully exploring the effects of aging on the human brain. Few reports have been found in studies of functional brain networks using functional near-infrared spectroscopy (fNIRS). Moreover, little is known about the feasibility of using fNIRS to assess age-related changes in brain connectomes. This study applied whole brain fNIRS measurement, combined with graph theory analysis, to assess the age-dependent changes in resting-state brain networks. Five to eight minutes of resting-state brain hemodynamic signals were recorded from 48 participants (18 young adults and 30 older adults) with 133 optical channels covering the majority of the cortical regions. Both local and global graph metrics were computed to identify the age-related changes of topographical brain networks. Older adults showed an overall decline of both global and local efficiency compared to young adults, as well as the decline of small-worldness. In addition, young adults showed the abundance of hubs in the prefrontal cortex, whereas older adults revealed the hub shifts to the sensorimotor cortex. These obvious shifts of hubs may potentially indicate decreases of the decision-making, memory, and other high-order functions as people age. Our results showed consistent findings with published literature and also demonstrated the feasibility of whole-head fNIRS measurements to assess age-dependent changes in resting-state brain networks.

## 1 Introduction

The human brain is organized in a set of networks, with anatomical brain regions involved in either individualized processing or integration with other brain regions, to accomplish different functions.^1^^,^^2^ Both anatomical and functional brain networks change in their properties as normal aging commences in humans. Some research studies have shown that the decline of gray matter in older individuals is most responsible for age-related changes in brain anatomy.^1^^,^^3^ Early studies have reported the preservation of small-world and economic brain characteristics in older adults,^1^ with a decrease of efficiency mostly in frontal and temporal cortical and subcortical regions.^4^ These findings explain well that, as people age, there is a decrease in their cognitive abilities, such as memory, attention, and concentration, which are functions controlled by the prefrontal cortex in humans. Recent studies have reported: (1) a decrease of functional connectivity in default mode network (DMN) and dorsal attention network;^5^ (2) an increase of functional connectivity in somatosensory and subcortical networks;^5^ (3) age-related effects on brain network connectivity;^6^ and (4) age-induced alterations in modularity and the number of hubs of the brain network.^7^ Understanding age-related alterations in human brain networks can help better understand the cognitive declines, guide early diagnosis of geriatric diseases, such as Alzheimer’s disease,^8^ Parkinson’s disease,^9^ and other dementias in older patients, and provide insight into effective treatments for these illnesses.

Such quantitative analyses, such as seed-based functional connectivity, independent component analysis (ICA), and graph theory analysis (GTA), have been proposed and utilized to investigate functional brain networks.^6^^,^^10^ In comparison, seed-based connectivity needs a predefined or prechosen seed region, whereas ICA is limited by its statistical nature that requires a large and low-noise data set with excellent neurological/neuroanatomical knowledge for correctly discriminating multiple artifact-driven, independent components from true functional networks.^6^ In the context of GTA, brain networks can be depicted as a graph with different anatomical and/or functional brain regions represented by nodes and with any interaction represented by links between each pair of brain regions.^2^ The nature of this graph-based approach allows us to examine the network connectivity among all brain regions/areas independently. In this way, both local and large-scale brain network features can be assessed efficiently by comprehending both temporal and spatial characteristics, with a relatively simple method. In particular, GTA has been successfully applied for the assessment of age-related changes in brain networks measured by structural and functional MRI,^2^^,^^11^ magnetoencephalography (MEG) and electroencephalography (EEG),^12^^,^^13^ and positron emission tomography (PET).^3^ In this paper, we chose GTA as our means to assess the age-related complex brain networks based on our whole-cortex, 133-channel, functional near-infrared spectroscopy (fNIRS) measurements.

It is well known that fNIRS is a noninvasive neuroimaging modality.^14^ It has been recently combined with GTA to successfully reveal the topographical organization of resting-state functional connectivity (RSFC) in the human brain.^15^^,^^16^ Zhang et al.^17^ reported distinct small-world features in the frontal cortical areas when the participants undertook deceptive actions with respect to their wakeful rest. Li et al.^18^ utilized GTA to reveal age-related changes in the anterior cortical regions. However, all the reported fNIRS-based GTA studies had a common deficiency: limited measurement channels (source–detector pairs) resulted in partial coverage of the brain networks on a human head. For example, 24 channels were used to investigate only the middle frontal and sensorimotor (SM) cortex,^17^ and 70 channels were able to cover only prefrontal, SM, and part of frontal-parietal (FP) cortical regions.^18^ While a total of 46 channels were employed to cover the entire human cortex, the placement or distribution of the channels was very sparse.^15^ Such a sparse-channel setup would fail to provide accurate or comprehensive connectomic information, confound the results because of lack of measurements from distal locations, and further limit the exploration of topographical connectivity among multiple cortical network systems.^15^

To overcome this obstacle and further extend the fNIRS brain imaging method into the brain network science, the current study utilized a 133-channel fNIRS system, which provided us with the excellent ability to cover the whole-cortical regions of the human brain, and to assess age-related changes in resting-state graphical brain networks. We hypothesized that (1) both young and older adults present distinct global network metrics and small-world features in the functional brain connectivity and (2) the reorganization of network hubs occurs in the older adults with respect to the younger group. This study aimed to prove our working hypotheses by performing 133-channel fNIRS measurements, leading to quantitative characterization of age-dependent changes in resting-state, whole-cortex graphical networks.

## 2 Methods and Materials

### 2.1 Participants and Data Acquisition

A total of 48 participants participated in this study, including 18 young adults [mean±standard deviation(SD)=26.5±2.5 years of age] and 30 older adults (mean±SD=73.3±7.5 years of age). All participants were right-handed with normal visual ability. No participants reported any known diseases, such as musculoskeletal, neurological, visual, or cardio-respiratory dysfunctions. The age range of the young adults was at or beyond the developmental/maturation phase of the prefrontal cortex,^19^ and that of the older adult group was at or beyond the documented declining phase of the gray matter density (i.e., at the age of 65).^20^ Written consent forms were signed by all participants before the experiment started. The study protocol was approved by the Institutional Review Board of the University of Texas at Arlington.

**Fig. 1:**
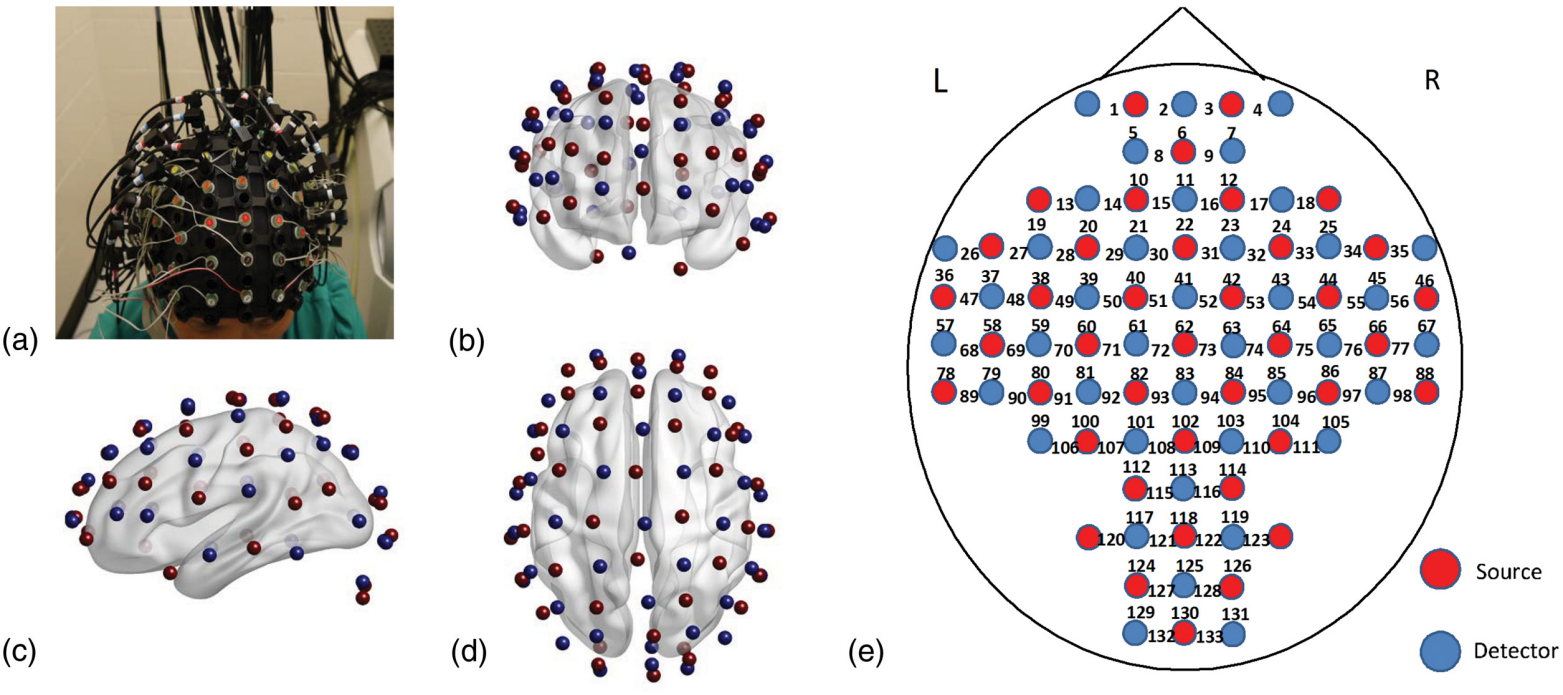
(a) A photo of the optode placement through a cap on a participant’s head. The optodes of 40 sources (red in the online version) and 40 detectors (blue in the online version) are marked and shown in (b) front view, (c) left-side view, and (d) top view. The location/place between each source–detector pair corresponds to a respective channel from 1 to 133, as labeled in (e). The (e) schematic illustration of the fNIRS 40-source and 40-detector opotodes, which were coregistrated and projected on a human brain template derived from the ICBM152 MNI space (see Sec. 2.2 for details).

In this study, we employed a continuous wave, 133-channel, high-performance, fNIRS system (LABNIRS, Shimadzu Corp., Kyoto, Japan), which consists of 40 sets of colocated NIR semiconductor lasers at three wavelengths (i.e., 780±5, 805±5, and 830±5  nm), and 40 photomultiplier tube detectors, to record cortical hemodynamic activity from each young and older adult. As shown in Fig. 1(a), an fNIRS cap can hold 40-source optodes and 40-detector optodes, and cover each participant’s head completely and symmetrically. The nearest source–detector separation was set to be 3 cm. With this optode geometry, a total of 133 channels (i.e., source–detector pairs) were formed [see Figs. 1(b)–1(e)], and only 3-cm source–detector channels were used. The fNIRS data were acquired at a sampling rate of 8.13 Hz.

After 40-source and 40-detector optodes were noninvasively and securely attached on each human participant’s whole head (see Fig. 1), she/he was instructed to sit comfortably in a quiet, light-dimmed room with eyes closed at wakeful resting state. All 133 channels simultaneously recorded fNIRS signals at three wavelengths across the entire head, over a period of 5 to 8 min, while the participants kept the sitting position with minimum body movements.

### 2.2 Optode Coregistration on a Human Brain Template

A coregistration procedure was followed, using a three-dimensional (3-D) digitizer that measured four cranial landmarks and all optodes from four randomly selected participants to estimate the fNIRS optode locations.^21^ Specifically, after placing the fNIRS optodes over each participant’s head, the four reference cranial landmarks (i.e., the nasion, left and right preauricular points, and vertex), light sources and detectors were measured using a 3-D motion tracking system (FASTRAK, Polhemus, Vermont, USA). The positions of cranial landmarks served as an affine transformation to convert the real-world stereotaxic coordinates of the optical optodes to the Montreal Neurological Institute (MNI) coordinates, which were used in a standard brain MRI atlas, as demonstrated by previous fNIRS studies.^22^^–^^24^ The space coordinates of 133 channels obtained by the 3-D digitizer were further coregistered in the ICBM 152 template.^25^ Then each of the brodmann areas (BA) probed by the fNIRS optodes was identified using the statistical parametric mapping for near infrared spectroscopy software package.^26^ A manual comparison of BA with an automated anatomical labeling atlas^27^ was performed to further classify or assign the 133 channels into five of the predefined large-scale parcellation network regions [fronto-parietal, default, SM, occipital (OC), and regions not included in any networks]. In particular, the coregistered positions (averaged over the four participants) of optodes were marked on a brain template (i.e., ICBM 152)^25^ as shown in Figs. 1(b)–1(d).

### 2.3 Data Processing

Time sequences of light intensity changes from 133 channels were recorded for all participants during a resting-state period (5 min for older adults and 8 min for young adults) at a sampling frequency of 8.13 Hz. The reason that we designed 5-min resting-state measurements (instead of 8 min) for older adults was to minimize the drossiness or sleepiness in the older population. It was reported that the 5-min resting-state fNIRS measurements were dynamically stable enough to give rise to reliable functional connectivity.^28^ Actually, to confirm the consistency between the RSFC derived from 5-min versus 8-min duration, we performed the same analysis using both time durations for the young participants. The results are presented in Appendix A.

All the raw data were visually inspected to reject motion artifacts and other large noise.^22^^–^^24^ A bandpass filter of 0.01 to 0.3 Hz was utilized to minimize the physiological noise generated by heartbeat and respiration.^24^ Moreover, to minimize confounding effects of superficial layers (i.e., the human scalp and skull) and overall global noise, a global autocorrelation process was sequentially applied to the channel-wise fNIRS data.^29^^,^^30^ Specifically, one global temporal profile G(t) was generated by averaging over 133-channel time sequences for each participant. An autocorrelation approach was performed between G(t) and the time series from each individual channel by calculating Pearson’s correlation coefficient, R. Any channel having a high correlation value of R with the global G(t) was considered to result from physiological noise or artifacts and was excluded for further data analysis. A threshold of |R|>0.2 was chosen to eliminate these channels based on previous suggestions.^31^

We calculated relative changes in concentrations of oxygenated hemoglobin (ΔHbO) and deoxygenated hemoglobin (ΔHbR) using the modified Beer–Lambert law.^32^^,^^33^ The sum of ΔHbO and ΔHbR gave rise to changes in total hemoglobin concentration (i.e., ΔHbT=ΔHbO+ΔHbR). In this study, ΔHbO signals were chosen to perform comprehensive network analysis and evaluate reproducibility of network metrics across participants and over time. Because most of the ΔHbR signals had relatively low intensities, we excluded them for further data analysis, as done in many other previous studies.^22^^,^^34^^–^^36^

### 2.4 Construction of Functional Brain Networks

**Fig. 2:**
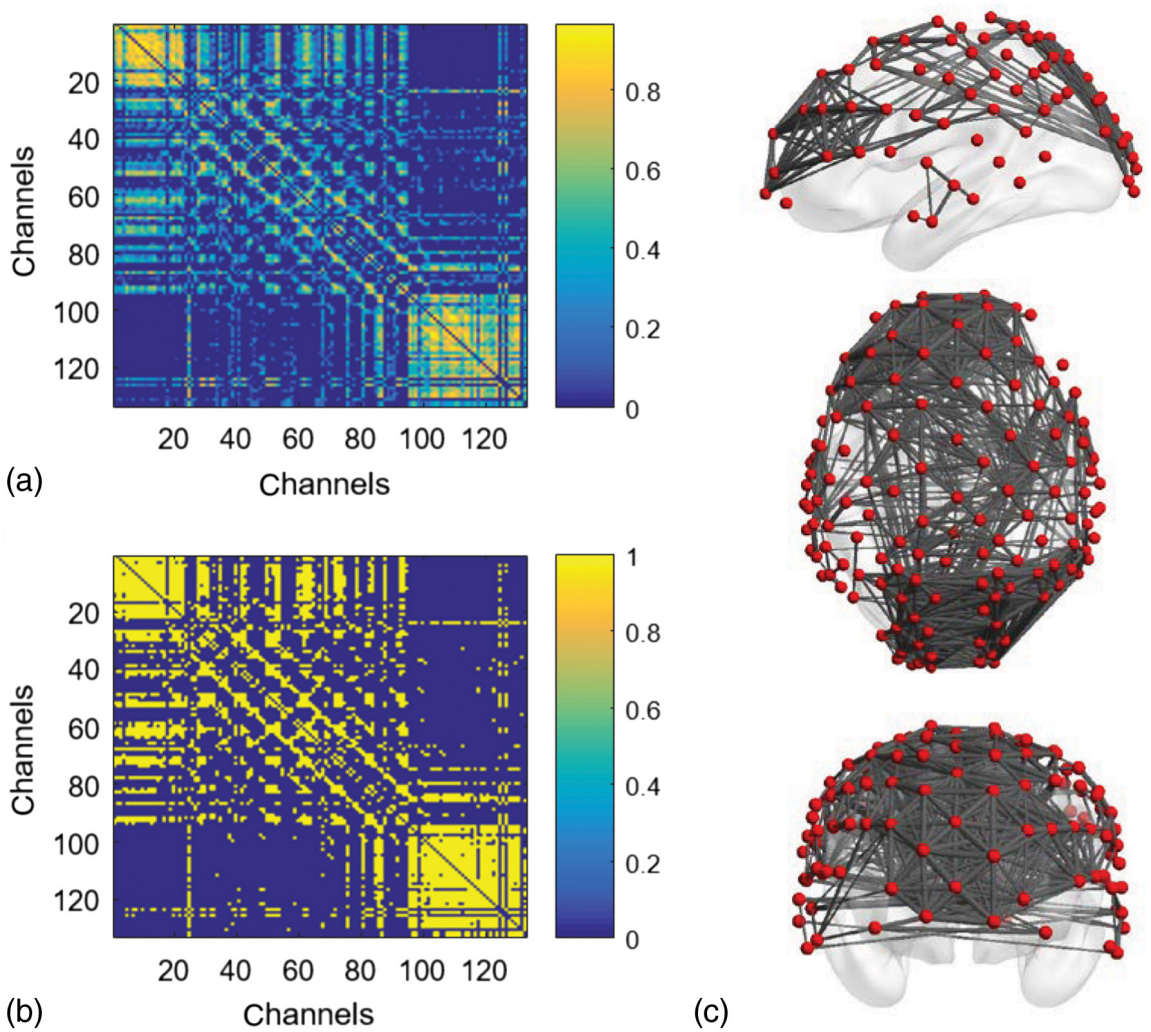
Construction of functional brain networks from fNIRS measurements. (a) A sample of adjacency matrix computed by cross correlation of resting-state cortical hemodynamic responses from one participant. (b) The corresponding binary matrix at the threshold of sparsity 0.3. (c) 3-D views (i.e., left-side, top, and front view) of the brain network derived from (b) at the sparsity of 0.3. Images were obtained using BrainNet Viewer software.^37^

Two major steps, namely, graph formation and network parameter quantification, are suggested in GTA.^6^^,^^10^ In this study, each channel represented one node in the brain network. Graph formation was then obtained by analyzing channel-wise or nodal ΔHbO signals and performing the cross correlation between each pair of the nodes to form a cross-correlation matrix (or adjacency matrix) [Fig. 2(a)]. The local and global graph parameters were then quantified from the cross-correlation matrix as the brain network characteristics.^10^

Former studies using fNIRS-GTA to investigate brain networks suggest that the brain functional connectivity could be represented by the correlations of brain hemodynamic changes or fluctuations among different brain regions.^15^^–^^17^^,^^38^ The same strategies were applied to nodal ΔHbO data during the resting-state period to establish the adjacency matrix for each participant. The cross correlation between each pair of nodal ΔHbO was then performed for the given time series; respective Pearson correlation coefficients (R) were computed to form a 133×133 adjacency correlation matrix [see Fig. 2(a)]. Note that the color (in the online version) in Fig. 2(a) denotes the values of correlation coefficient. For example, blue color indicates a correlation coefficient smaller than 0.5; orange color represents a correlation coefficient larger than 0.5. The adjacency matrix was further converted into a binarized matrix by setting a threshold. The correlation coefficient between nodes i and j (i=1,2,…133; j=1,2,…,133) was set to 1 if the correlation value was larger than the given threshold, and 0 otherwise [see Fig. 1(b)]. The two nodes were defined as connected if the binarized correlation value was equal to 1, and there was no functional connection between two nodes if the binarized correlation value was equal to 0.

The connecting line (or edge) between two connected nodes was used to graphically mark functional connectivity across the whole human brain/cortex. In principle, different selections of thresholds on cross-correlation coefficient will result in a different binarized matrix. In this study, we applied different thresholds to the adjacency matrix to obtain a sequence of binary matrixes. Specifically, the sparsity-based approach was utilized as suggested by Niu et al.^15^ The sparsity (S) for a fixed graph is defined as the number of current existing edges in this graph, divided by the maximum possible number of edges in the current graph. In this study, the range from 0.05 to 0.5 (i.e., 0.05<S<0.5, interval=0.01) was chosen to be the standard threshold sequence as reported in a previous study.^3^ Then the threshold sequence was applied to the adjacency matrix to generate a total of 45 binarized network matrices for each participant. Figure 2(b) shows an example from one participant’s data, revealing a spatial representation of nodes and edges from one binary matrix with a given/fixed threshold (S=0.30). To illustrate 3-D representation of the binary matrix, left-side, top, and front views of the brain network were obtained using BrainNet Viewer software^37^ together with the ICBM152 brain template [Fig. 2(c)]. Red dots (in the online version) represent the nodes, whereas gray/black lines between two nodes represent respective network edges or connections.

### 2.5 Graph Theory Analysis

Based on the adjacency matrices, we further quantified the resting-state brain network parameters using GTA. In general, graphical metrics for functional brain networks are calculated based on global and local network characteristics.^10^ The global network metrics include such “small-world” properties as: (1) clustering coefficient ($C_{p}$); (2) characteristic path length ($L_{p}$); (3) normalized clustering coefficient ($\gamma =C_{p}^{\text{real}}/C_{p}^{\text{random}}$); (4) normalized characteristic path length ($\lambda =L_{p}^{\text{real}}/L_{p}^{\text{random}}$); and (5) small worldness (σ=γ/λ). Briefly, $C_{p}^{\text{real}}$ is the average of clustering coefficients over all nodes in a network, quantifying the extent of local group of a network.^39^^,^^40^ $L_{p}^{\text{real}}$ is defined as the average of the shortest path lengths between any pair of nodes in the network, quantifying the capability of parallel information propagation within a network.^41^ Also, $C_{p}^{\text{random}}$ and $L_{p}^{\text{random}}$ are the mean clustering coefficient and characteristic path length of matched random networks that preserve the same number of nodes, edges, and degree distribution as the real network.^42^ A real network would be considered small-world if γ>1 and λ≈1. Another global metric we studied^6^ was global efficiency ($E_{g}$). Furthermore, for the local graphical metrics, we focused on the hub information such as: (1) nodal degree ($N_{i}$); (2) nodal efficiency ($E_{\mathrm{nod}}$); and (3) betweenness centrality ($N_{\mathrm{bc}}$). Detailed descriptions of these graph metrics are included in Appendix B.

### 2.6 Statistical Analysis

Statistical analysis was performed to quantify interregional correlations for bilateral nodal regions in both young and older adult groups. Totally, 133 pairs of brain nodal regions were compared by the z-values (Z) that were obtained after Z transformation of the Pearson correlation coefficients (R) in order to meet the statistical normality requirement. To further test the hypothesis of between-group differences in the graphical metrics, pairwise t-tests were performed to compare the young and older adults in $E_{g}$, $L_{p}$, $C_{p}$, γ, λ, and σ. A criterion of p<0.05 was selected.

## 3 Results

### 3.1 Global Network Characteristics and Small-Worldness

#### 3.1.1 Global features in young and older adults

**Fig. 3:**
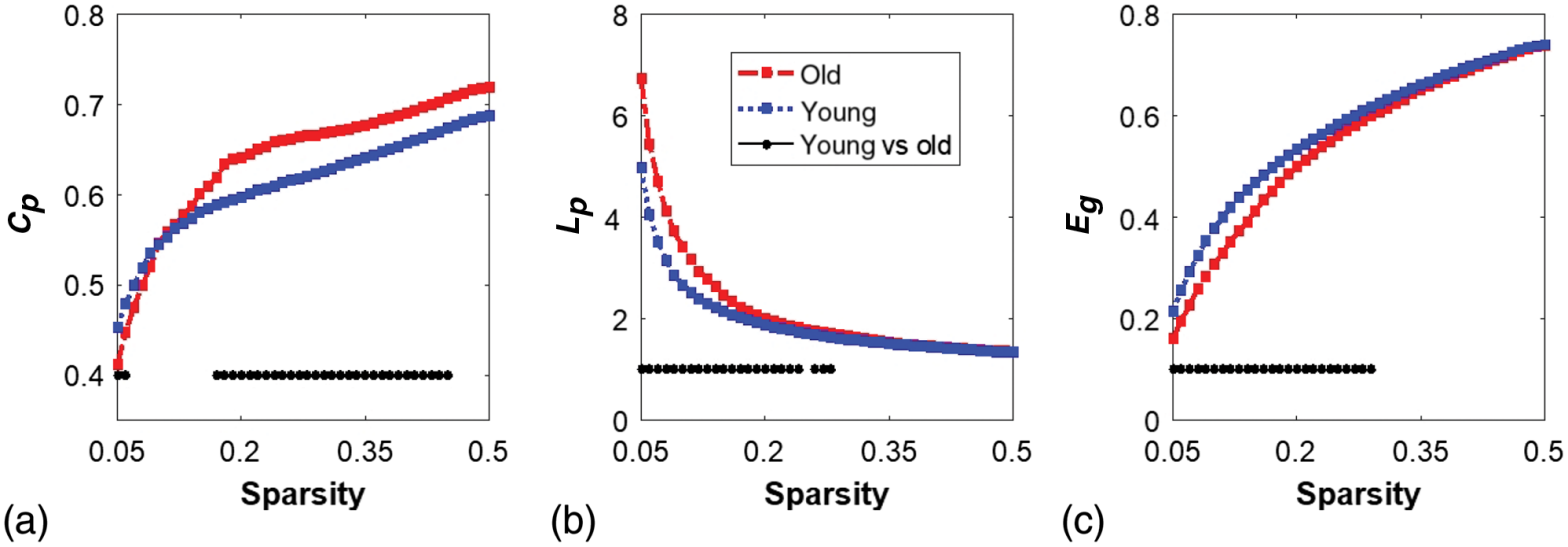
Global characteristics of resting-state brain networks derived from GTA. (a)–(c) The sparsity-dependent clustering coefficient ($C_{p}$), shortest path length ($L_{p}$), and global efficiency ($E_{g}$) values quantified from young adults (blue in the online version) and older adults (red in the online version). The black dots on the bottom of each panel mark the sparsity ranges where significant differences in respective network parameters exist between two groups. Specifically, older adults have a larger $C_{p}$ at the lower sparsity range (0.18 to 0.46) and a larger $L_{p}$ in the lower sparsity range (0.05 to 0.29), whereas young adults reveal a larger $E_{g}$ in the sparsity range (0.05 to 0.30).

We quantified and compared the global network and/or small-world features of the brain networks between young and older adults. The respective values of $C_{p}$, $L_{p}$, and $E_{g}$ of both groups, are shown in Figs. 3(a)–3(c). Two-sample t-tests for each of the global brain network parameters were performed at each sparsity value from 0.05 to 0.5, as suggested by a previous study.^3^ Note that global features of the brain networks from the young group were plotted together with those from the older adults for easy comparison.

The group-level age-related changes in $C_{p}$, $L_{p}$, and $E_{g}$ were observed. Overall results illustrated that, compared to young adults, older adults showed higher $C_{p}$ and $L_{p}$, but lower $E_{g}$. Since we considered the data at each sparsity (or threshold) to be independent, there was no need to run ANOVA tests. We chose the two-sample t-test with unequal sample size and two-tailed analysis, having a criterion of p<0.05 selected. Specifically, older adults revealed a significantly larger $C_{p}$ value within the sparsity range between 0.17 to 0.45 [see Fig. 3(a)]. Meanwhile, older adults exhibited a significantly larger $L_{p}$ value than young adults within the lower sparsity range between 0.05 to 0.29 [see Fig. 3(b)]. There was no significant difference between two groups when S>0.3. Young adults, on the other hand, had a significantly larger global efficiency $E_{g}$, than older adults within the lower sparsity range of 0.05 to 0.30 [see Fig. 3(c)]. Both groups shared the similar $E_{g}$ in the higher sparsity range (S=0.31 to 0.50) and indicated the trend of convergent to 1 as the sparsity increased. Our findings are consistent with a previous study,^3^ which investigated resting-state brain networks in 115 young adults (average age=35) and 110 older adults (average age=54) using PET. Both their results and ours clearly demonstrated a decline of global efficiency with an increase in clustering coefficient in older adults.^3^

#### 3.1.2 Small-world features in young and older adults

**Fig. 4:**
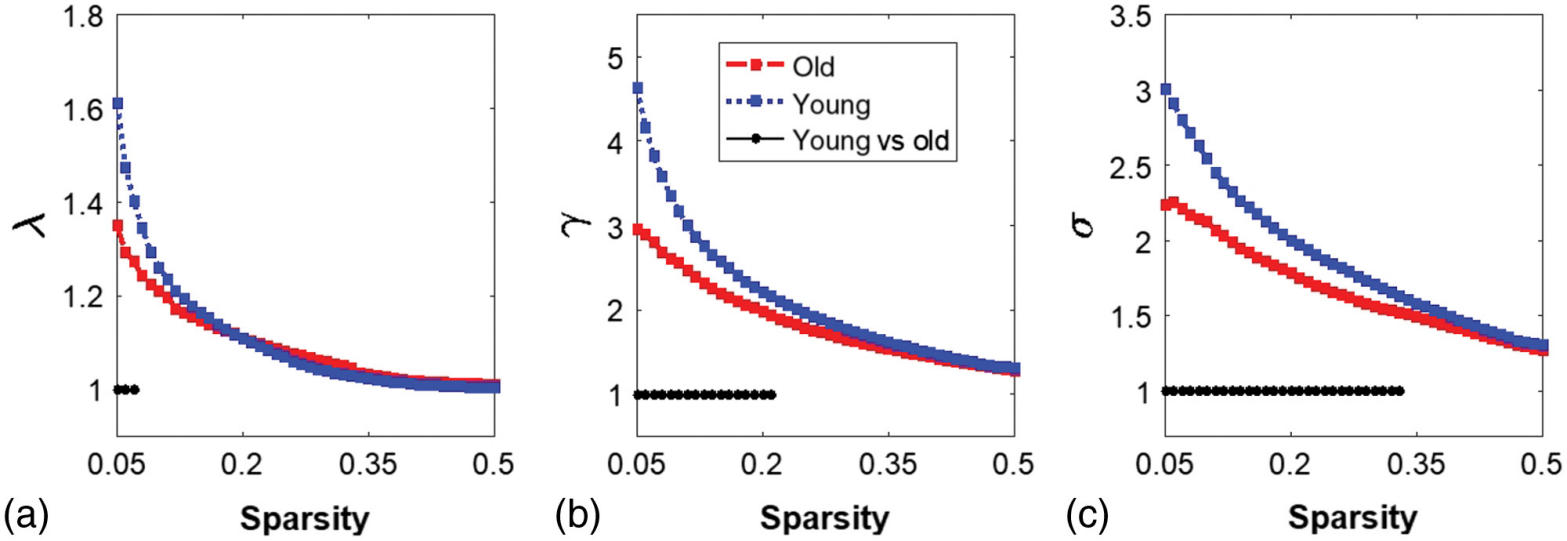
Small-worldness features in young and older adults. (a)–(c) The normalized characteristic path lengths (λ), normalized clustering coefficient (γ), and small-worldness (σ) quantified from young adults (blue in the online version) and older adults (red in the online version), respectively. The black dots on the bottom of each panel indicate significant differences of each parameter between the two groups within the given sparsity range for λ(0.05<S<0.07), γ(0.05<S<0.22), and σ(0.05<S<0.34).

Functional networks of the human brain have small-world characteristics;^10^^,^^43^ a real network would be considered small-world if $\gamma (=C_{p}^{\text{real}}/C_{p}^{\text{random}})>1$ and $\lambda (=L_{p}^{\text{real}}/L_{p}^{\text{random}})\approx 1$. It means that, compared to random networks, a true human brain network has a larger clustering coefficient and an approximately identical shortest path length between any of two nodes in the network. Figures 4(a)–4(c) show the normalized characteristic path length λ, normalized clustering coefficient γ, and small worldness σ, taken from the two age groups, respectively. It is clear that λ values in the two groups were approaching 1, with sparsity 0.05<S<0.5 [Fig. 4(a)], and so did γ within 0.3<S<0.5 [Fig. 4(b)]. At a wide range of sparsity, both groups had σ>1 [Fig. 4(c)], which implies prominent small-world properties. Regarding age effects, the results demonstrated that the young adults had significantly better normalized clustering ability and small-worldness of the brain networks than the older group, based on larger values of γ(0.05<S<0.22) and σ(0.05<S<0.34).

### 3.2 Local Graphical Parameters

**Fig. 5:**
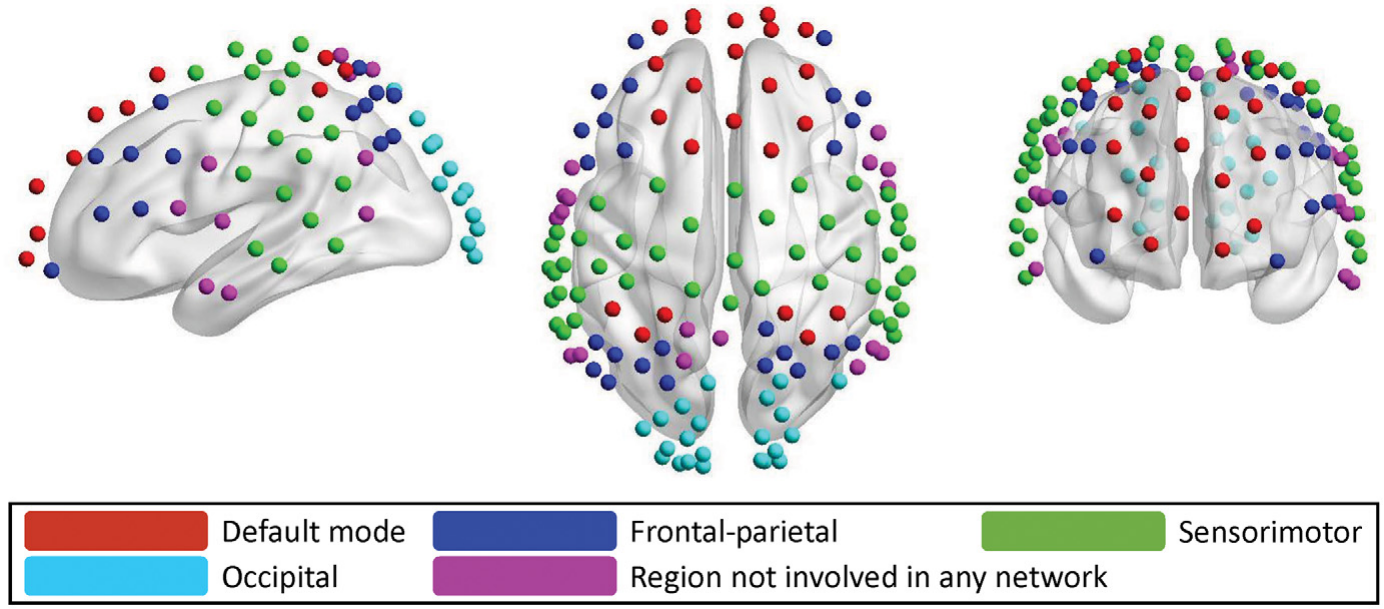
Illustration of 133 optodes or nodes that were identified into five predefined networks to match predefined functional brain networks. The color-coded networks (only seen in the online version) are: DMN (red, n=23), FP network (blue, n=25), SM network (green, n=45), OC network (azure, n=22), and region NI in any network (purple, n=18). All the networks are manually defined with careful guidance by BA atlas.

**Fig. 6:**
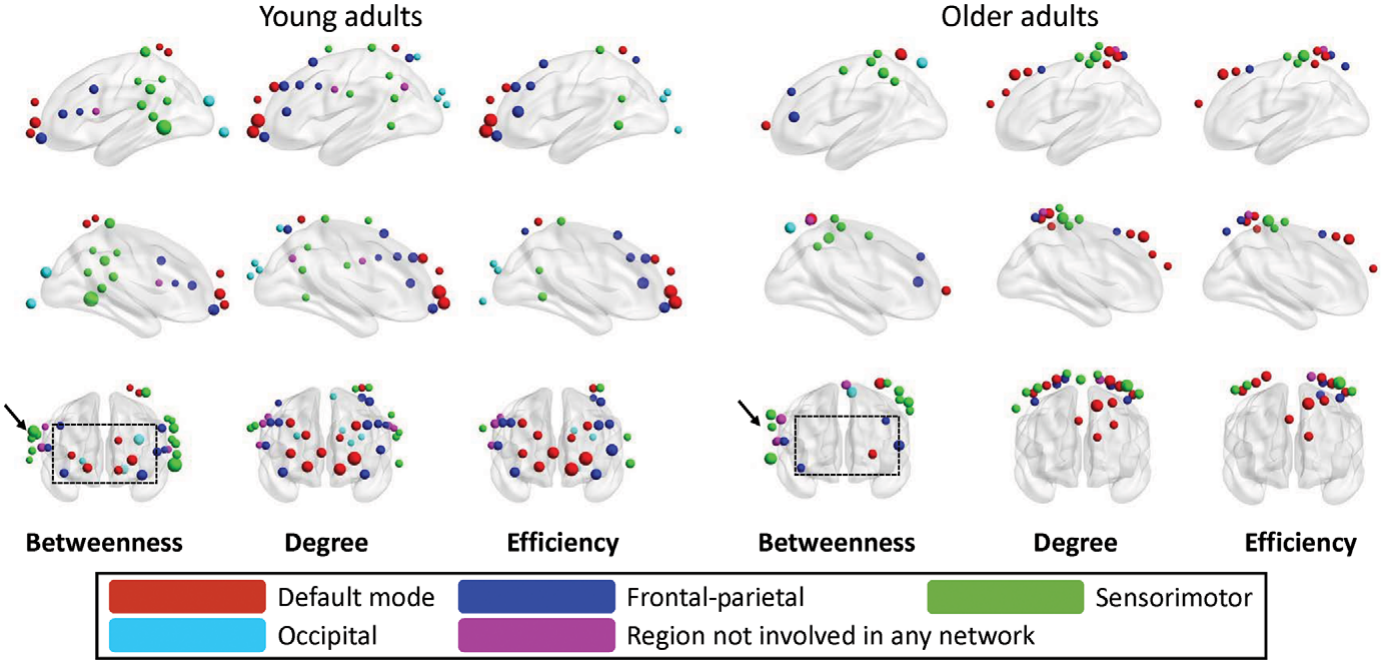
Hubs compared between young and older adults. For all three of betweenness centrality ($N_{\mathrm{bc}}$), nodal degree ($N_{i}$), and nodal efficiency ($E_{\mathrm{nod}}$), (i) red dots represent the hubs within the DMN, (ii) blue dots represent the hubs within the FP network, (iii) green dots represent hubs within the SM network, (iv) azure represent hubs within the OC network, and (v) purple dots represent hubs that is NI in any network. The size of the dots represents the strength of three parameters.

We quantified such local graphical parameters in this study as nodal degree ($N_{i}$), nodal efficiency ($E_{\mathrm{nod}}$), and betweenness centrality ($N_{\mathrm{bc}}$). These three nodal metrics are well-accepted parameters with distinct emphasis to reflect local network hub properties. The nodal degree is a simple measure of the number of connections at each node. The nodal efficiency represents the cost of information transfer through that node. The betweenness centrality is a measure of centrality in a graph based on shortest paths. The nodal metrics were constructed at a sparsity threshold of 0.15, as suggested by a previous study^3^ in order to ensure that the networks of both young and older adult groups had the same number of nodes and edges. Then network hubs were selected for each of the three nodal parameters (i.e., $N_{i}$, $E_{\mathrm{nod}}$, and $N_{\mathrm{bc}}$), with respective values larger than 1 SD of the corresponding average values over all nodes.^15^^,^^16^ Figure 6 demonstrates left-side, right-side, and front views of the hubs determined from the young adults and older adults for all three nodal metrics [i.e., betweenness centrality ($N_{\mathrm{bc}}$), degree ($N_{i}$), and efficiency ($E_{\mathrm{nod}}$)]. As indicated by Niu et al.,^15^ the betweenness centrality is considered as the major reference for the hub measurements. In addition, we also manually partitioned the 133 channels into five network regions, as shown in Fig. 5, based on the guidance of BA atlas as mentioned in Sec. 2.2. These five networks are: (i) DMN (red); (ii) FP network (blue); (iii) SM network (green); (iv) OC (azure); and (v) channels not involved (NI) in any network (purple).

The number of hubs corresponding to the network locations in both age groups was summarized in Table 1, with corresponding illustrations in Fig. 6. In the older adult group, a total of 18, 26, and 19 hubs were identified under betweenness, nodal degree, nodal efficiency, respectively. In contrast, a total of 33, 40, and 30 hubs were correspondingly identified in the young adult group. These results revealed an overall decline of the total hub number from 103 to 63 (∼39% reduction), in the older adult group. Correspondingly, Fig. 6 shows three types of hubs for both age groups [i.e., betweenness centrality ($N_{\mathrm{bc}}$) nodal degree ($N_{i}$), and nodal efficiency ($E_{\mathrm{nod}}$)], in five brain networks. The size of the dots represents the strength of three parameters.

**Table 1:** Hubs in young and older adults.

	Young adults				Older adults			
	Btw	*R* (%)	Degree	Efficiency	Btw	*R* (%)	Degree	Efficiency
DMN	7	21	10	10	2	11	11	10
FP	7	21	12	10	4	22	5	3
SM	13	40	8	4	8	44	9	5
OC	3	9	5	3	1	6	0	0
NI	3	9	5	3	3	17	1	1
Total	33		40	30	18		26	19

Because nodal betweenness quantifies how important a node is within a network, we paid special attention to this metric. With close inspection of Fig. 6, we observed that 10-11 hubs (with all colors) appeared in the middle and dorsolateral frontal regions in the young adult group (see the dashed box in the left-most column of Fig. 6, with the notation of “betweenness”); only 4 hubs showed up, relatively unilaterally, within the similar region in the older adult group (see the dashed box in the middle of Fig. 6, also with the notation of “betweenness”). Furthermore, the hubs in the SM region seemed unilateral in the older adults (see an arrow near the dashed box in Fig. 6), in contrast to a bilateral pattern presented in the young adult group (see another arrow near the dashed box in the left-most column of Fig. 6).

Next, still focusing on nodal betweenness, we noted that the number of hubs shown in Table 1 seemed to shift from DMN to other networks in the older adults. To be more quantitative, we defined a ratio ($R_{k}$), between the number of hubs in the specific network (k) and the total number of hubs (only for nodal betweenness), where k represents DMN, FP, SM, OC, and NI, respectively. All respective values of $R_{k}$ were calculated and are listed in Table 1 for both groups. One clear and predominant distinction between the two groups is that the $R_{\mathrm{DMN}}$ ratio of the older adults reduced by ∼50% (from a ratio of 21% to 11%), as compared to the younger group, meaning that the number of nodal betweenness hubs in DMN was reduced, or significantly shifted to other brain networks, when people age.

## 4 Discussion

### 4.1 Whole-Head fNIRS Measurements

Our study, for the first time, applied a whole-head fNIRS approach to assess age-related changes of RSFC in the human brain or cortex. The GTA was chosen as the assessment tool for the quantification of functional brain networks. Both global and local graphical metrics were computed; comprehensive comparisons between young and older adults were also conducted. With 80 optodes (40 for sources and 40 for detectors), we were able to cover most of the cortical regions of each human participant. This whole-head coverage ensured the cerebral hemodynamic measurements with both abundant distal and local connectomic information. Our findings for age-related RSFC changes were consistent with what have been reported by MEG and fMRI. Specifically, this study successfully observed that: (1) the functional networks in both age groups held well small-world (i.e., σ>1) characteristics, efficiently providing high-information processing with low connection cost; (2) older adults decreased small-world features [Fig. 4(c)] and global efficiency [Fig. 3(c)], with increased global clustering coefficient and shortest path length [Figs. 3(a) and 3(b)], revealing gradual progression of brain network/connectivity changes along aging; (3) normal aging resulted in much decline (∼40%) in functional connectivity hubs over the whole-cortical regions, and also led to a decrease in the hub symmetry; and (4) an approximate 50% decrease of nodal betweenness hubs in the DMN was observed in the older adult group. (This significant decrease of frontal connectivity could be associated with the decline of short-time memory and of integration of information.)

One advantage of whole-head fNIRS shown in this study was the ability to identify specific brain networks based on the hemodynamic fNIRS measurements. Specifically, we were able to identify five different brain networks (see Fig. 5) by the whole-head optode setup with 23, 25, 45, 22, and 18 nodes in each predefined network, respectively. This classification enabled us to assess the brain networks through cortical topography, with both enough distal measurements and detailed local records. Previously, while the feasibility of using fNIRS to assess graphical brain networks with good reproducibility was demonstrated, most of those studies were not able to associate functional brain connectivity with specific/known brain networks.^15^^,^^16^ This problem stemmed from sparse placement of the optodes on the participant’s head, so the coverage was limited to partial regions of the brain, such as prefrontal cortex^18^ and prefrontal-motor cortex.^17^ While this study demonstrated consistent findings on global network properties with those reported in Refs. 15 and 18 using either sparse optodes or partial coverage of the head, the whole-cortical measurements would provide us with more accurate and comprehensive nodal information and local network parameters than the other two studies. The latter cases may cause misleading conclusions on local network metrics due to a lack of distal network features.

### 4.2 Age-Effect on Global Topographical Metrics

Another innovative aspect of this study was that it has clearly revealed age-related changes in the resting-state brain networks at the whole-cortex level. While the age effect of the human brain networks has been observed and demonstrated by both fMRI and PET,^3^^,^^44^ there is little work on this topic by optical methods. In this current study, we found and investigated the DMN; FP network; SM network; OC network; and other regions in both young adults (24 to 28 years of age) and older adults (65 to 82 years of age). Different from previous fNIRS studies, we utilized the whole-head opotode setup to be able to cover and investigate the entire cortical networks instead of having only partial coverages, such as only interrogating the prefrontal cortex.^18^ Our findings are consistent with previous studies.^3^^,^^45^^,^^46^ First, we showed higher global efficiency in young adults than in older adults. It is believed that the anatomical alterations during aging play a role for these network changes, especially after age of 65.^47^

Second, our results also revealed a difference in clustering coefficients between the two age groups. Similar findings from previous reports^3^^,^^4^ have suggested that a possible reorganization of the frontal network occurs as people age. It is also suggested that the information processes are less economical in older adults, especially in the frontal and temporal cortical and subcortical regions.^4^ One of the current findings different from our previous report^18^ is that we found an increase of characteristic path length, instead of no change, with an increase in age. This difference could possibly result from the benefit of covering the whole head of each human participant with 133 channels that interrogated not only local brain regions (i.e., prefrontal in our previous study), but also long-distant functional associations. The agreement between our current findings and those using fMRI and PET^3^^,^^4^^,^^48^ strongly supports the observation/conclusion that normal aging leads to the loss of long-distance connections and interconnected hubs that decrease the network economics.

Finally, we also observed a significant decrease in small-worldness in older adults, which was in good agreement with previous studies.^2^^,^^3^^,^^44^ One fMRI study^4^ with the measurement of 11 older adults (age = 66.5 years) in a resting state reported that normal aging impaired economic performance of small-world brain networks. Similar findings were also suggested by a PET study^3^ with 113 young (age = 36.5 years old) and 110 older (age = 56.3) adults. In addition to these consistent findings, we observed that the small-worldness in older adults were still significantly higher than the random network in the sparsity ranges given [Figs. 4(a) and 4(b)], revealing that an older brain still preserved economical performance of the network, although it was lower than the younger group.

### 4.3 Age-Effect on Local Topographical Metrics: Reduction and Reorganization of Hubs

One noticeable observation for the local hubs was the reduction of the total number from 33 in young adults, to 18 in older adults (quantified by betweenness centrality), and this mostly happened in the default mode (7 versus 2), and was relatively obvious in the FP (7 versus 4) networks. Specifically, the nodal betweenness in the default mode regions (such as middle frontal and superior frontal regions) was diminished in the older adults. Because the hubs play the central role of integrative processes and communication,^17^ the overall decline of number of hubs suggests the loss of information-processing centers in the aged brain. The long-distance connections, especially the between-network connections, such as frontal-SM and frontal-OC, are expected to decrease, as shown in our age group data. A recent fMRI study, with 24 older (59 to 74 years of age) and 21 young (18 to 26) adults, also suggests similar findings that DMN and FP control network in older adults have fewer common hubs compared with younger brains.^6^ We can speculate that the long-distant connections are more vulnerable with aging.^6^ Unfortunately, we could not find any optical data at the present time to substantiate these findings.

In addition, although the total number of hubs had significantly decreased in the older group, the ratio of FP hubs over the total hubs was kept the same, and the ratios of SM, OC, and undefined hubs were slightly increased in the older group. This interesting finding indicates that the older brain is trying to preserve certain numbers of hubs in primary processing centers, such as SM and OC areas. In sum, the reduction of hubs and changes of symmetrical characteristics should lead to a behavioral shift in the older brain. The abundancy of the network hubs in the primary information-processing region (such as SM networks) and the reduction of hubs within networks supporting higher-level cognitive functions (such as default and FP networks) coincide with the phenomenon that older adults often show the decline of cognitive functions.^6^^,^^7^

### 4.4 Further Discussion on Resting-State Brain Network and Connectivity

Resting state of the human brain is an uncontrolled condition, and it can be studied according to temporal or frequency features of the brain,^49^^,^^50^ or according to hemodynamic or electrophysiological characteristics of the brain.^49^^–^^51^ A large amount of studies in all four aspects have been reported, with a handful of literature only on fNIRS-derived resting-state connectivity.^15^^,^^18^^,^^38^^,^^52^^,^^53^ Different resting-state networks were obtained when different analysis methods were used. In this paper, we utilized the GTA to analyze HbO-derived brain networks and then determine differences in whole-cortical brain networks at wakeful rest between young and older adults. Note that the network properties reported in this paper could be different if other age-related neurophysiological conditions are considered, such as effect of neurovascular coupling. However, a few recent studies reported that normal aging does not cause significant changes in neurovascular coupling derived from blood-oxygen-level-dependent signals.^54^^,^^55^ If one wishes to include/study electrophysiology-related brain networks at rest, dual-mode (fNIRS-EEG or fMRI-EEG) brain imaging measurements and corresponding data analysis would be necessary.

### 4.5 Limitations of the Study and Future Work

In any study of this type, there are often a few limitations that should be noted. First, it is known that the NIR light can penetrate through the human scalp, skull, and a portion of gray matter, but the light could not go deeper than 3 cm below the human scalp.^56^ The fNIRS measurements taken from the scalp surface gather a majority of the information from the gray matter in the cortical regions; thus this study reveals global and local graphical metrics for only cortical connectivity and networks. Deeper layers of the human brain, such as those in subcortical areas, are “out of reach” by the fNIRS optical measurements, regardless of whole-head or partial brain measurements.

Second, there is a trade-off between covering a larger portion of the head versus the time-cost of the experimental setup. A whole-head optode setup can provide an abundancy of collected data from the whole head, but it increases a significant amount of time for placing the optodes and adds much more weight to the participant’s head, which is more likely to cause the participant too uncomfortable, motion artifacts, and even more physiological noises. To overcome these drawbacks, future work can include development of: (1) quicker or more efficient ways to place optodes through hair; (2) a light-weight helmet with a thinner and more flexible layer; (3) a fiber-supporting frame/stand to reduce the setup time and the weight on the participant’s head. Nevertheless, this study demonstrated the feasibility of using fNIRS to assess resting-state functional cortical networks and their age-related changes.

Third, a potential confounding factor to the findings resulted from the possibility that the participants, especially the older adults, could go to non-rapid-eye-movement (NREM) sleep during the measurements, which could give rise to certain spatial changes of the resting-state network during the transition from wakefulness to light NREM sleep, as reported by Refs. 57–59. Thus inclusion of several key EEG channels characteristic for identifying sleep states is suggested for future studies.

Finally, in this study, we did not record the ethnicity information in the demographic data. The young adult participants were recruited from graduate students on campus of the University of Texas at Arlington. The older adults were recruited from local community of senior centers, with majority being Caucasian population. To avoid potential bias on the results caused by ethnic difference, we will collect ethnicity information and consider the corresponding effect in future studies.

## 5 Conclusion

In this present investigation, we introduced/combined graphical theory analysis with the whole-head fNIRS measurements to assess age-related changes in the resting-state functional brain networks. We successfully proved two working hypotheses, namely, we quantified and identified distinct global and local graphical metrics of the cortical brain networks between young and older adults, and revealed reorganization of network hubs in the older adults compared to the younger group. A decrease in global efficiency and increases in the clustering coefficient and shortest path length were observed as characteristic features of older participants, which reflected brain network changes due to aging. In addition, we observed the age-related decline of major functional connectivity hubs in the default mode network, which might underlie the decline of short-time memory and integration of information. Our present findings are also consistent with the literature. All of these strongly demonstrated the feasibility of whole-head fNIRS measurements to assess age-dependent changes in resting-state brain networks, which could have a variety of clinical applications in the near future.

## Figures and Tables

**Fig. 7:**
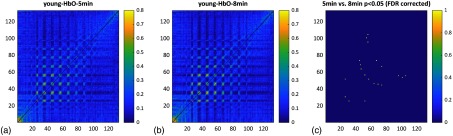
Adjacency matrices derived from (a) 5-min, (b) 8-min duration, and (c) their comparison across the young adult group.

## Appendix A: Effects of 5-min Versus 8-min Duration on Functional Connectivity

To confirm the consistency between the resting-state functional connectivity derived from 5-min versus 8-min duration, we performed a group-level network-based statistical analysis within the young adult group. The time series data were separated into two groups: (a) within the first 5 min and (b) whole period of 8 min. Panels (a) and (b) in Fig. 7 show the group-averaged adjacency matrices derived from 5-min and 8-min duration across the young adult group. The third panel (c) indicates the functional connectivity pairs that are significantly (marked by dots) and nonsignificantly different (marked by background color) between the two time-duration cases. The statistical analysis used a criterion of p<0.05 with false discovery rate correction. We observed a total of 20 pairs (dots) that were significantly different versus a total of 17,556(=133×132) pairs within the adjacency matrix. These results illustrated or confirmed that effects of 5-min versus 8-min duration of data acquisition were minimal on quantification of functional connectivity since only 0.11%(=20/17,556) of total optode pairs were statistically affected by these two different time durations.

## Appendix B: Brain Network Metrics

For a defined network or graph N, there are n nodes and k edges; the global and local network metrics are the output of the GTA.^2^ Using the GRETNA program, we computed the following graph metrics in our study.^10^^,^^37^

The following parameters are global network metrics:

1. *Global efficiency* ($E_{\mathrm{glob}}$):
  $$E_{\mathrm{glob}}=\frac{1}{n(n-1)}\sum_{i\neq j\in N}\frac{1}{d(i,j)}.$$
  This is the average inverse shortest path length d(i,j) between two nodes i and j. n is the number of nodes in the network N.
2. *Clustering coefficient* $C_{P}$:
  $$C_{p}=\overset{n}{\underset{i=1}{\sum }}\frac{2N_{i}}{c_{\mathrm{nod}}(i)[c_{\mathrm{nod}}(i)-1]}.$$
  This is the sum of number of existing connections of the node’s neighbors divided by the number of all their possible connections. $N_{i}$ denotes the number of existing connections among the neighbors of node i, and $c_{\mathrm{nod}}$ represents the number of edges that are connected to node i.
3. *Characteristic path length* ($L_{p}$):
  $$L_{p}=\frac{1}{n(n-1)}\sum_{i\neq j\in G}d(i,\;j).$$
  This is the minimum number of edges that link any two nodes of the network. d(i,j) is the shortest path length between node i and node j.
4. *Normalized clustering coefficient* (γ):
  $$\gamma =C_{p}^{\mathrm{real}}/C_{p}^{\mathrm{rand}}.$$
  This is the mean of all clustering coefficients over all nodes in a network. $C_{p}^{\mathrm{real}}$ and $C_{p}^{\mathrm{rand}}$ are the clustering coefficient in a real network and random networks.
5. *Normalized path length* (λ):
  $$\lambda =L_{p}^{\mathrm{real}}/L_{p}^{\mathrm{rand}}.$$
  This is the average of the shortest path lengths between any nodes of the network. $L_{p}^{\mathrm{real}}$ and $L_{p}^{\mathrm{rand}}$ are the characteristic path length in a real network and random networks. Not that current study averaged 100 random networks in the calculation.
6. *Small-worldness* (σ):
  σ=γ/λ
  The normalized characteristic clustering coefficient divided by the normalized characteristic path length. A network is considered small-world if σ>1.
7. *Nodal degree* ($N_{i}$):
  $$N_{i}=\underset{j\neq i\in G}{\sum }a_{ij}.$$
  This is the number of edges linked directly to a particular node. $a_{ij}$ is the i’th row and j’th column element in the adjacency matrix.
8. *Nodal efficiency* ($E_{\mathrm{nod}}$):
  $$E_{\mathrm{nod}}=\frac{1}{n-1}\sum_{j\neq i\in G}\frac{1}{d(i,j)}.$$
  It is defined as the inverse of the harmonic mean of the minimum path length between a particular node and all other nodes in the network. d(i,j) is the shortest path length between node i and node j.
9. *Nodal betweenness centrality* ($N_{\mathrm{bc}}$):
  $$N_{\mathrm{bc}}=\underset{m\neq i\neq n\in G}{\sum }\frac{\delta_{mn}(i)}{\delta_{mn}},$$
  This is the number of shortest paths between any two nodes that run through a particular node (i). $\delta_{mn}$ is the total number of shortest paths from node m to node n and $\delta_{mn}(i)$ is the number of shortest paths from node m to node n that pass through node i. A high $N_{\mathrm{bc}}$ denotes large impacts of this node on the information flow over the whole network.
